# ELMER v.2: an R/Bioconductor package to reconstruct gene regulatory networks from DNA methylation and transcriptome profiles

**DOI:** 10.1093/bioinformatics/bty902

**Published:** 2018-10-26

**Authors:** Tiago C Silva, Simon G Coetzee, Nicole Gull, Lijing Yao, Dennis J Hazelett, Houtan Noushmehr, De-Chen Lin, Benjamin P Berman

**Affiliations:** 1 Department of Biomedical Sciences, Center for Bioinformatics and Functional Genomics, Cedars-Sinai Medical Center, Los Angeles, CA, USA; 2 Department of Genetics, Ribeirão Preto Medical School, University of São Paulo, Ribeirão Preto, Brazil; 3 Bioinformatics Research & Early Development, Roche Sequencing Solutions, Belmont, CA, USA; 4 Department of Neurosurgery, Henry Ford Hospital, Detroit, MI, USA; 5 Department of Medicine, Cedars-Sinai Medical Center, Los Angeles, CA, USA

## Abstract

**Motivation:**

DNA methylation has been used to identify functional changes at transcriptional enhancers and other cis-regulatory modules (CRMs) in tumors and other disease tissues. Our R/Bioconductor package *ELMER* (Enhancer Linking by Methylation/Expression Relationships) provides a systematic approach that reconstructs altered gene regulatory networks (GRNs) by combining enhancer methylation and gene expression data derived from the same sample set.

**Results:**

We present a completely revised version 2 of *ELMER* that provides numerous new features including an optional web-based interface and a new Supervised Analysis mode to use pre-defined sample groupings. We show that Supervised mode significantly increases statistical power and identifies additional GRNs and associated Master Regulators, such as *SOX11* and *KLF5* in Basal-like breast cancer.

**Availability and implementation:**

*ELMER* v.2 is available as an R/Bioconductor package at http://bioconductor.org/packages/ELMER/.

**Supplementary information:**

Supplementary data are available at *Bioinformatics* online.

## 1 Introduction

Motivated by the identification of transcription factor binding sites (TFBSs), enhancers and other cis-regulatory modules (CRMs) from DNA methylation data in tumor samples (Berman *et al.*, 2012; Hovestadt *et al.*, 2014; Johann *et al.*, 2016), and the strong association between DNA methylation and target gene expression in tumors (Aran *et al.*, 2013; Aran and Hellman, 2013), we previously developed an R/Bioconductor package *ELMER* (Enhancer Linking by Methylation/Expression Relationships) to infer regulatory element landscapes and GRNs from cancer methylomes (Yao *et al.*, 2015). ELMER version 1 has been adopted by other groups (Dhingra *et al.*, 2017; Malta *et al.*, 2018; Mishra and Guda, 2017), and remains the only publicly available software tool to use matched DNA methylation and expression profiles to reconstruct TF networks (reviewed in Teschendorff and Relton, 2017). Other tools such as TENET (Rhie, 2016) and RegNetDriver (Dhingra *et al.*, 2017) have incorporated ELMER principles and code into cancer network analysis.

**Fig. 1.**
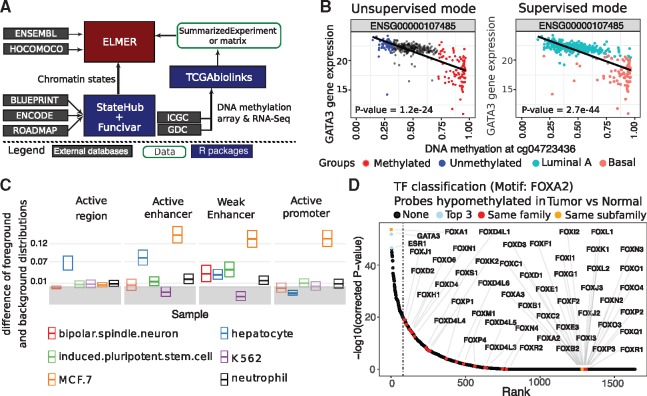
(**A**) ELMER architecture, showing external data sources (gray) and Bioconductor packages (blue). (**B**) Association of enhancer probe methylation and expression of the nearby *GATA3* gene, showing TCGA breast cancer sample groups used in the *Unsupervised* versus *Supervised* analysis modes. In *Unsupervised* mode, the 20% of samples with the lowest (blue) and highest (red) methylation levels are compared; in *Supervised* mode, the predefined Luminal A (blue) and Basal-like (red) tumors are compared. (**C**) StateHub chromatin state enrichment analysis for 1076 regulatory elements identified in the *Unsupervised* analysis. (**D**) Master Regulator analysis for the top motif in the *Unsupervised* analysis, *FOXA2.* All TFs are ranked by their correlation with methylation changes of distal probes within 250 bp of a *FOXA2* binding motif. Colored dots indicate the top 3 most anti-correlated TFs (*FOXA1*, *GATA3* and *ESR1*), and all TFs classified in the same family as *FOXA2*

We present here a substantially re-written ELMER v. 2 (Fig. 1A) that implements new features and improvements including: (i) support for Infinium HM450 or EPIC arrays and RNA-seq using the gold-standard MultiAssayExperiment (MAE) data structure, (ii) integration with our TCGABiolinks package (Colaprico *et al.*, 2015) for cohort selection and data importing from the NCI Genomic Data Commons (Grossman *et al.*, 2016), (iii) integration with our TCGABiolinksGUI tool (Silva *et al.*, 2018) to run ELMER via a web-based interface, (iv) output of all results in a single interactive HTML file include all data tables, figures and source code, (v) adoption of software engineering best practices including unit testing and better exception handling, (vi) annotation of cell-type specific chromatin context for resulting genomic elements and (vii) a new *Supervised* mode where the user can explicitly define sample groups for comparison. In this brief Note, we highlight several of these new features by analyzing TCGA Breast Cancer data to identify molecular subtype-specific networks. A complete description of new methods and features, along with computational benchmarking, is presented in the Supplementary Methods and Notes (Supplementary Figs S1–S16 and Supplementary Tables S1–S5). ELMER v. 2 has been publicly available starting with v. 2.2.7 in Bioconductor Release 3.6 (October 2017). Complete result reports for the BRCA analyses are available in the Supplementary Material and at http://bit.ly/ELMER_reports.

## 2 Feature highlights

### 2.1 Supervised versus Unsupervised mode

ELMER first identifies Differentially Methylated CpGs (DMCs) occurring at distal (non-promoter) probes (Step 1), then searches for downstream gene targets for each DMC (Step 2), and finally identifies Master Regulator TFs based on enriched binding motifs and TF expression (Step 3), as shown in Supplementary Figure S1. ELMER v. 1 identified DMCs by comparing methylation in all cancer versus non-cancer samples, while the subsequent steps used correlation between methylation and expression in the *n*% of tumors with the most extreme methylation values (by default, *n* = 20). The rationale was that any particular GRN might only be altered in a subset of tumors with a specific molecular phenotype, which would not always be known *a priori.* While 20% was an arbitrary definition, we found this to be a useful exploratory strategy given the heterogeneity of cancer molecular phenotypes.

In ELMER v. 2, we continue to support this original *Unsupervised* strategy. However, we have found many practical use cases where the group structure is known in advance, and a *Supervised* search strategy is preferable. This is especially true for “case–control” experimental designs such as treated versus untreated samples. The major difference is that in *Supervised* mode, all samples must be contained in one of the two comparison groups, whereas *Unsupervised* mode still uses only the *n*% most extreme. Furthermore, this subset of samples with the most extreme methylation values changes from one genomic locus to the next.

To compare *Supervised* versus *Unsupervised* modes, we used ELMER v. 2.4.3 to analyze TCGA BRCA (Breast Invasive Carcinoma) data (Supplementary Figs S2–S15 and Supplementary Tables S2–S3). When considering enhancer-gene pairing, *Supervised* mode had greater statistical power (Fig. 1B), and identified more enhancer-gene pairs overall when molecular subtypes were pre-defined using the PAM50 molecular subtypes (Ciriello *et al.*, 2015) (Supplementary Fig. S3). Specifically, *Supervised* mode not only re-identified most of the results obtained by *Unsupervised* mode, but also generated many additional subtype specific enhancer-gene pairs. This comparison suggests that while *Unsupervised* mode can serve as a useful exploratory tool when sample subtype is unknown *a priori*, *Supervised* mode offers greater statistical power when sample subtype is pre-defined.

While it is very difficult to directly assess the false positive rates of *Supervised* versus *Unsupervised* analyses, we gained insight into the question by comparing ELMER-predicted enhancer-gene links to pairs identified using PolII looping (ChIA-PET) in Luminal type MCF7 cells (Li *et al.*, 2012). This analysis showed that while all of the Luminal-specific *Supervised* analyses produced pairs that were enriched in ChIA-PET loops (compared to randomized ELMER data), the pairs from the *Unsupervised* analysis were more strongly enriched based on both Precision and Recall values (Supplementary Fig. S8). For heterogeneous patient samples composed of multiple subtypes, it thus appears that *Unsupervised* and *Supervised* analyses can offer complementary merits, with *Unsupervised* analysis displaying a higher false negative rate, but a lower false positive rate. It is recommended to run both *Supervised* and *Unsupervised* analyses, as we demonstrated here, to gain maximum insight. This approach is discussed more below in the context of the Master Regulators identified.

### 2.2 Functional interpretation of chromatin states

While ELMER v.1 was limited to analyze only probes overlapping known enhancers, ELMER v.2 analyzes *all* distal probes, and thus it is now important to provide a functional interpretation of the resulting regions. We perform a chromatin state enrichment analysis using states automatically downloaded from the http://StateHub.org database, a publicly-available resource that integrates histone modification and other publicly-available epigenomic data for over 1000 different human samples (Coetzee *et al.*, 2018). Enrichment of these states is calculated against a randomly sampled background set drawn from the same distal probe set used as input. We used ELMER v.2 to perform this state enrichment analysis for the BRCA dataset, yielding insights into the cell-type specificity of the genomic regions identified (Fig. 1C and Supplementary Fig. S6). The strongest enrichment was for active enhancer and promoter states having cell-type specificity for MCF7, a Luminal Breast Cancer cell line.

### 2.3 Motif enrichment analysis and identification of Master Regulator TFs

The final step of ELMER identifies enriched TF binding motifs within candidate regulatory regions, followed by correlation with TF expression to identify upstream Master Regulators (Supplementary Fig. S1). ELMER v. 1 used a hand-curated selection of 145 TF motifs, which were grouped into binding domain families manually. We re-implemented these sections in ELMER v. 2 to use publicly available databases for these steps, making the package more comprehensive and easier to update in future versions. ELMER v. 2 uses 771 human binding models from HOCOMOCO v11 (Kulakovskiy *et al.*, 2018). Each of these is associated with one or more of 1639 transcription factors defined in Lambert *et al.* (2018), which are grouped into 82 different binding domain families and 331 sub-families using the TFClass database (Wingender *et al.*, 2018). We use the Fisher’s exact test and Benjamini-Hochberg multiple hypothesis correction to compare the frequency of each motif flanking the positive CpG probes to a background defined by all distal probes on the array, plotting the top hits as odds ratios with 95% confidence intervals (Supplementary Fig. S15).

For each enriched motif, we then calculate a mean DNA methylation value for all probes having a motif instance within ±250 bp, and correlate this value to each of the 1639 TFs in our database. This helps to distinguish between different members of the same TF family, which often have nearly indistinguishable binding motifs. For instance, in the BRCA analysis, the most highly enriched motif corresponded to *FOXA2*, but this Master Regulator (MR) analysis showed the likely family member to be *FOXA1* (Fig. 1D), which has been extensively validated as a MR in luminal subtypes of breast cancer (Meyer and Carroll, 2012; Nakshatri and Badve, 2009).

**Fig. 2.**
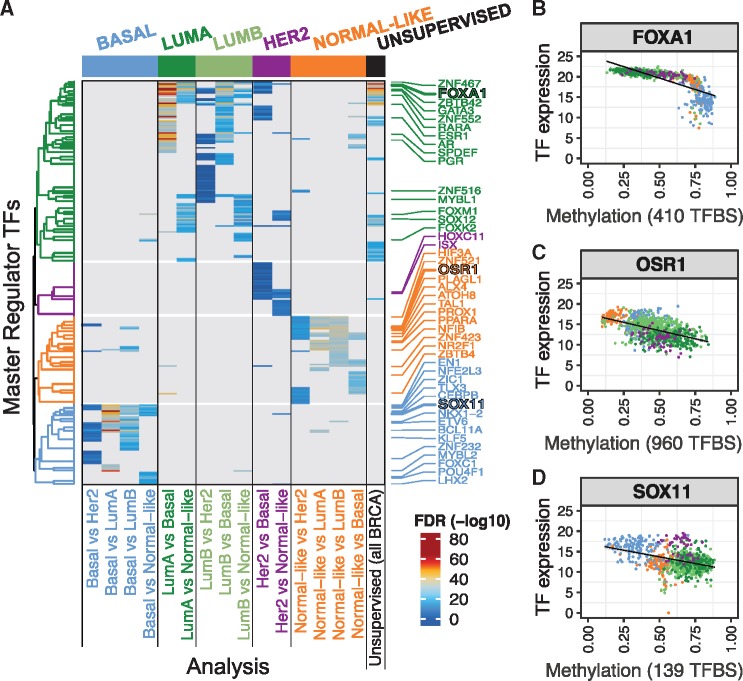
(**A**) List of all Master Regulators TFs identified in pairwise *Supervised* analyses between all PAM50 subtypes (left 15 columns) and an *Unsupervised* analysis (the right-most column). Each row is a Master Regulator TF, with expression vs. TFBS methylation FDR values color-coded in the corresponding analysis. TFs were clustered based on binary values (Jaccard dissimilarity), and four TF clusters were identified. TFs that were ranked among top five most significant hits were highlighted on the right. (**B–D**) Scatter plots showing TFBS probe methylation and expression of example TFs from different subtypes: *FOXA1* from Luminal (B), *OSR1* from Normal-like (C), and *SOX11* from Basal-like (D)

In order to directly compare the results of *Supervised* and *Unsupervised* modes, we performed a *Supervised* analysis for each pair of known PAM50 molecular subtypes (Ciriello *et al.*, 2015) (Fig. 2, Supplementary Table S3). Luminal-specific analyses successfully identified almost all of the MR TFs obtained by the *Unsupervised* analysis. More importantly, *Supervised* modes identified many additional MR TFs. For example, the Basal-specific analyses identified several factors that have been recently been described as functional in BRCA, including *SOX11* (Shepherd *et al.*, 2016) and *KLF5* (Ben-Porath *et al.*, 2008).

## 3 Conclusions and future directions

ELMER v. 2 has been substantially re-written based on Bioconductor standards and user needs. The new *Supervised* mode significantly improves the comparisons of two homogeneous groups, such as treated versus untreated, mutant versus wildtype, etc. For heterogeneous groups, we showed that *Unsupervised* and *Supervised* analyses can have complementary strength. Showcasing TCGA BRCA data, we used PAM50 (which was originally defined by unsupervised clustering of tumor expression data) for subtype definitions, but any multi-omic unsupervised clustering method can used, depending on what data types are available.

In addition to the new *Supervised* mode, our improved TF analysis identified additional known and novel Master Regulators candidates in TCGA BRCA analyses. ELMER v. 2 has only been tested on data from Illumina methylation arrays, which cover only 5-15% of all enhancer regions based on whole-genome bisulfite sequencing (WGBS). While *ELMER* does not currently support WGBS due to lack of sufficient test data, the number of WGBS datasets is quickly growing, and we expect the same basic ELMER approach will scale well in the future to take advantage of this more comprehensive data type.

## Funding

The project was funded by the Cedars-Sinai’s Samuel Oschin Comprehensive Cancer Institute, by the São Paulo Research Foundation (FAPESP) (2016/01389-7 to T.C.S. & H.N. and 2015/07925-5 to H.N.), by the NIH/NCI Informatics Technology for Cancer Research (1U01CA184826 to B.P.B., D.J.H & S.G.C) and Genomic Data Analysis Network (1U24CA210969 to B.P.B & T.C.S) programs, as well as NIH/NCI grant R01CA190182 to D.J.H.

*Conflict of Interest*: none declared.

## Supplementary Material

bty902_Supplementary_Methods
